# Path Tracking Control of Field Information-Collecting Robot Based on Improved Convolutional Neural Network Algorithm

**DOI:** 10.3390/s20030797

**Published:** 2020-01-31

**Authors:** Yili Gu, Zhiqiang Li, Zhen Zhang, Jun Li, Liqing Chen

**Affiliations:** 1 College of Engineering, Anhui Agricultural University, Hefei 230036, Chinaahnd1468732752@ahau.edu.cn (J.L.); 2 Anhui Province Engineering Laboratory of Intelligent Agricultural Machinery and Equipment, Hefei 230036, China

**Keywords:** information collection robot, control, machine vision, corn rows, path tracking

## Abstract

Due to the narrow row spacing of corn, the lack of light in the field caused by the blocking of branches, leaves and weeds in the middle and late stages of corn growth, it is generally difficult for machinery to move between rows and also impossible to observe the corn growth in real time. To solve the problem, a robot for corn interlines information collection thus is designed. First, the mathematical model of the robot is established using the designed control system. Second, an improved convolutional neural network model is proposed for training and learning, and the driving path is fitted by detecting and identifying corn rhizomes. Next, a multi-body dynamics simulation software, RecurDyn/track, is used to establish a dynamic model of the robot movement in soft soil conditions, and a control system is developed in MATLAB/SIMULINK for joint simulation experiments. Simulation results show that the method for controlling a sliding-mode variable structure can achieve better control results. Finally, experiments on the ground and in a simulated field environment show that the robot for field information collection based on the method developed runs stably and shows little deviation. The robot can be well applied for field plant protection, the control of corn diseases and insect pests, and the realization of human–machine separation.

## 1. Introduction

Accompanying the development of technologies in artificial intelligence and navigation, robots are increasingly being designed and applied to agricultural science, which is considered a most challenging area of human–computer interaction [1]. Recently, many scholars at home and abroad have conducted research on the structures of agricultural robots. Regarding a plant protection robot, its movement mechanism can be mainly divided into two types: a wheel type [2] and a track type [3,4]. Both have their own adaptive environment, respectively. Different moving mechanisms require different chassis designs, and the traction, steering and obstacle crossing are important factors that determine the performance of the robot [5,6]. To respond to the different functional requirements for the robot, the design of different structures are needed, and many scholars have carried out the research on the grasping mechanism of the robot, including the design and development of a mechanical arm for a transplanter to process paper can seedlings [7], and the design of a stable and reliable grabbing mechanism [8] for some agricultural product bags, such as tight packing, large deformation and easy damage. Concurrently, some researchers also designed and analyzed the end actuator [9,10]. So far, certain achievements have been made in the structural design of agricultural robots in grasping, moving and other motions. 

Agricultural robots can achieve stable work in the operation process, in addition to fulfilling the functional requirements in structure, it is also of great importance to accurately identify the working environment. The acquisition of working environment images mainly depends on a vision sensor [11]. A Kinect V2 camera in the vision sensor has a wide range of applications for its low price and strong robustness [12]. The vision system, as an important part of a robot, can be applied to fruit picking and other tasks [13]; vision transmission of up, down, left and right movements of the sensor can be controlled by the motor, so as to establish a vision navigation system of an agricultural robot with a variable vision field [14]. The recognition of the collection image by the vision sensor can be directly processed by an algorithm [13], or the image can be trained by a convolution neural network [15,16]. Generally, a diagnostic model trained with data under limited conditions may not involve situations that are not observed during training. To this end, a new type of deep adversarial convolutional neural network can be used [17]. Through picture recognition, the robot can detect the obstacles [18], then avoid the obstacles and a desired path can be planned [9,19], so as to realize the robot‘s moving and turning in the field environment.

As a crop widely planted in China, corn often suffers from plant diseases and insect pests in the middle and late stages of its growth, mainly because it has a high-stalk type, growth higher in the middle and late stages, and lacks sufficient light as it is often shaded by leaves and branches. Such conditions make it difficult to be recognized by conventional visual technology. Additionally, the rows of corn cultivation in China are spaced by 60 cm, adding more difficulty for interline movement, when compared with ordinary machinery. To be able to detect pests and other information in corn growth in real time, the authors designed a robot to collect crop growth information in the field. A target detection method, which is based on Faster R-CNN [20] and transfer learning on the convolutional network model of VGG-16 [21], is proposed to realize the robot’s recognition of inter-row information, with dual-motor drive control technology used to achieve interline movement by the robot.

**Figure 1:**
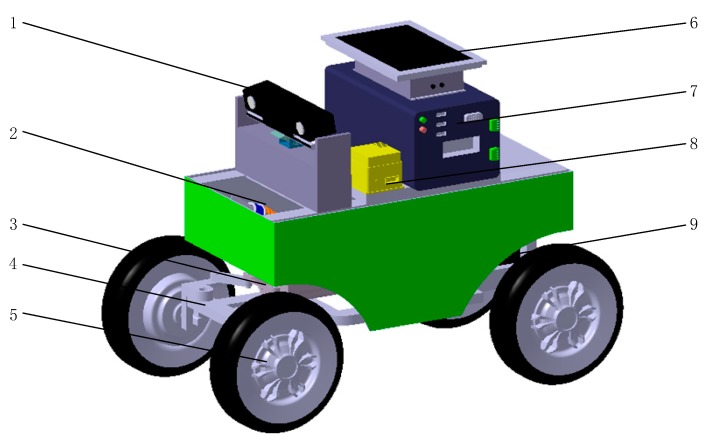
Structure diagram of field information collection robot: **1.** Kinect camera; **2.** Damping spring; **3.** Stepper motor; **4.** Steering mechanism; **5.** Hub motor; **6.** Host computer; **7.** Industrial personal computer; **8.** Inertial navigation; **9.** Lithium battery pack.

A three-dimensional model of the robot for field information acquisition, based on CATIA software, is shown in Figure 1. The robot is mainly composed of four parts: a steering module, an image acquisition module, a power module and a drive module. While working, the path video image collected by the camera is transmitted to an industrial computer for processing and analysis. The computer communicates with the microprocessor STM32F103C8T6 through the serial port, and transmits the navigation path information obtained from the processing and analysis to both brushless DC and step motor drivers. The driving force of the robot is provided by a hub motor, with the pulse with modulation (PWM) regulated by the brushless DC driver and the steering of the whole machine controlled by the stepper motor driver; the stepper motor and the steering mechanism are driven by the gear rack for precise steering. The power for the above control system is provided by a 48-volt lithium battery. 

**Figure 2:**
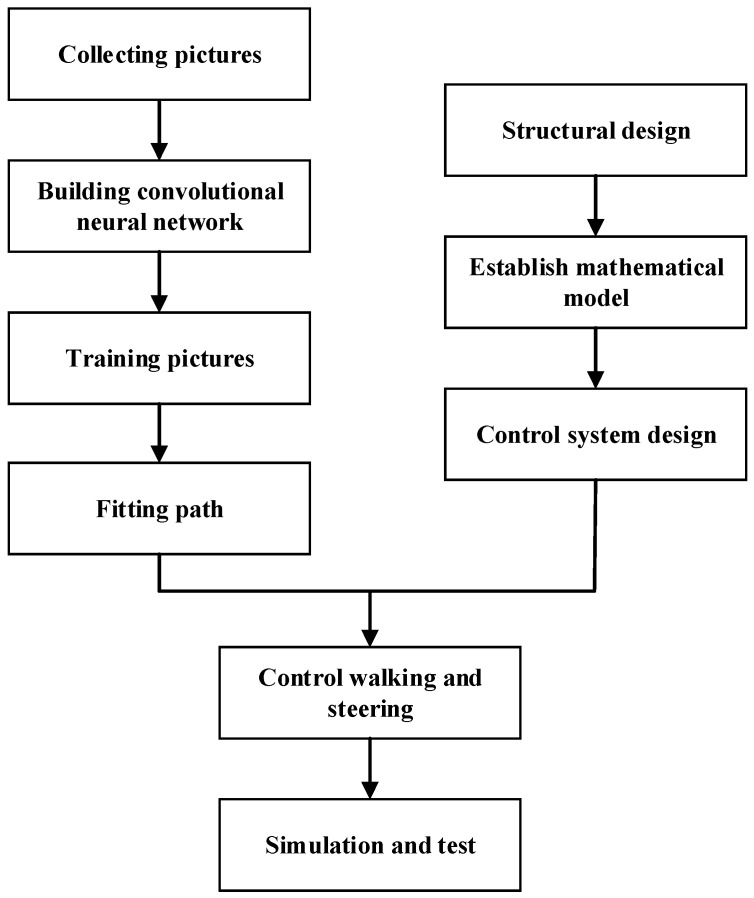
Flow chart of robot field path recognition.

The overall structure of the article is shown in Figure 2: 

## 2. Establishing Convolutional Neural Network

### 2.1. Image Acquisition

**Figure 3:**
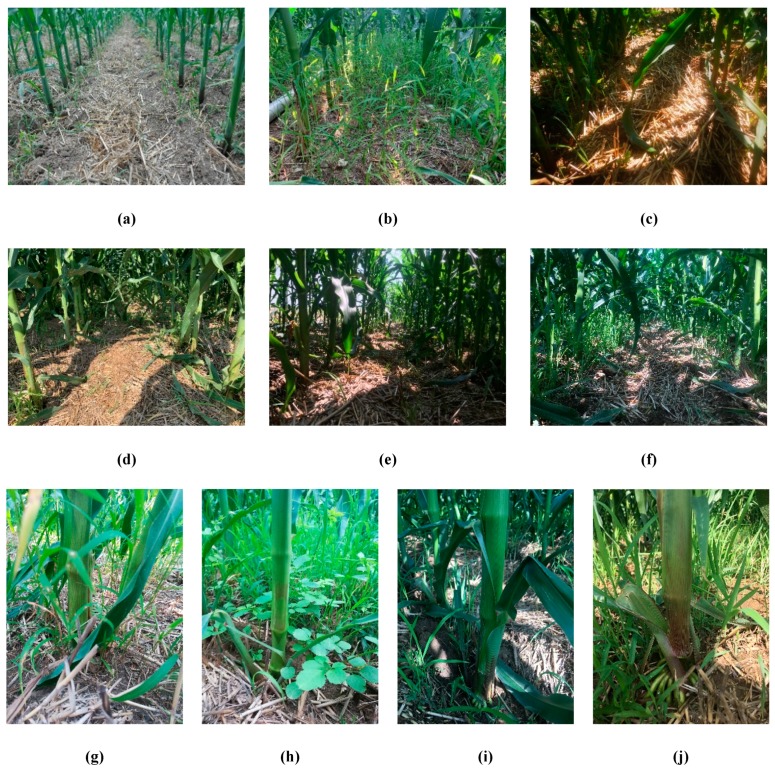
Sample images in different environments. (**a**) Ideal environment; (**b**) Excessive weeds; (**c**) Insufficient illumination; (**d**) Disordered branches and leaves; (**e**) Branches, leaves and light effects; (**f**) Weeds, branches and leaves are covered, and the light is insufficient; (**g**) Branches and leaves; (**h**) Weeds; (**i**) Branches and leaves block light; (**j**) Branches, leaves and weeds cover the rhizome.

First, the camera was calibrated based on Zhang Zhengyou’s checkerboard method [22] to obtain its inherent parameters and distortion coefficients. Then, 5420 corn rhizome images were collected at different heights, angles and lighting conditions, as shown in Figure 3. Among them, 550 pictures were collected at the trefoil stage, 620 at the jointing stage, 2110 at the male tasseling stage, and 2140 at the mature stage. During the first two periods the pictures were taken from the top, while in the second two they were collected in all directions flush with the corn rhizome. The pictures in each period were collected in three intervals, that is, morning, noon and evening in equal number. The rhizome pictures collected during the tasseling and mature stages were used for training, including single-rhizome ones and those selected from multiple rhizomes. It was found that the lack of light, due to leaf interference and excessive weeds (a manifestation of the complexity of the interline environment), poses a difficulty for traditional image processing methods to accurately identify corn rhizomes.

### 2.2. Image Training

The convolutional neural network is a deep learning model that specializes in processing grid-like data. It consists of the layers for input, convolution, pooling, full connection, and output. The convolutional layer is composed of multiple feature surfaces, each feature surface made up of multiple neurons, each of which is connected to a local area of the previous feature surface through a convolution kernel. The convolutional neural network takes the original image as an input and performs a convolution operation on the convolution layer with the feature map of the previous layer and a convolution kernel. The convolution result is mapped by the activation function to form the feature map of the next layer. The pooling layer mainly reduces the dimension of the feature map between consecutive convolutional layers, maintains the translation invariance of the data to a certain extent, and decreases parameters and calculations in the network. The fully connected layer is located at the end of the structure of the convolutional neural network model, with each neuron in it fully connected to all the neurons in the previous layer. The layer can integrate the class-specific local information in the convolutional or the pooling layer. As a classifier output image, the number of neurons in the fully connected network structure is the same as the output of the convolutional layer.

A visual geometry group (VGG) network is one of the widely used convolutional neural network (CNN) models, which has a strong expansibility and a simple structure. The main purpose of this model is to study the influence of convolution network depth on the accuracy of large-scale image recognition. Here, the convolution network model of VGG-16 is used as the source pre-training model of an object detector, while the data set of corn rhizomes is used for training. The convolution core was set to 3 ∗ 3.

**Figure 4:**
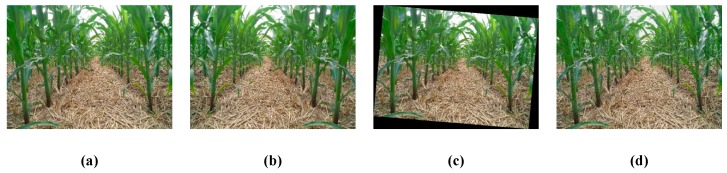
data enhancement method. (**a**) original image; (**b**) mirrored image; (**c**) affine transformed image; (**d**) noisy image.

To shorten the training time, the difference of gaussian (DOG) pyramid model [23], based on Gauss kernel, was used to reduce the image of a single corn rhizome to the same scale to form a data set. Considering the sake of parameter accuracy and the normal work of the network model, we used rotation transformation, image transformation and addition of noise to the collected image as data enhancement, as shown in Figure 4. The enhanced data set was divided into a training set and a test set in proportion 7:3. Considering the global learning rate of region-CNN (R-CNN) set at $1*10^{-5}$, the network was trained. The output of CNN in this paper is the position of the corn rhizome in the picture.

### 2.3. Model Accuracy Evaluation

**Figure 5:**
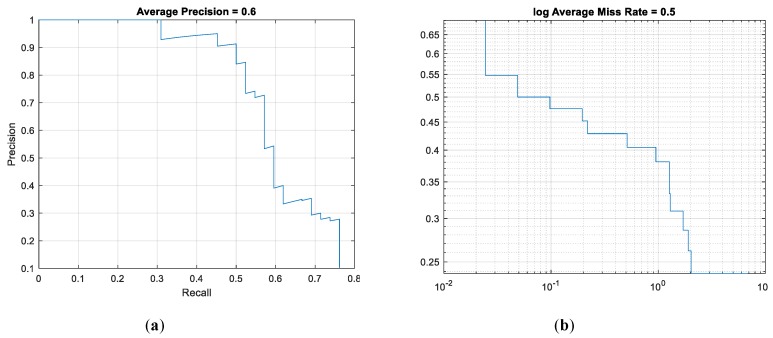
Model accuracy evaluation curve. (**a**) accuracy and recall curve; (**b**) error rate curve.

To further verify the accuracy of the object detection model, it is necessary to determine the corresponding indicators for evaluation. Recall rate, accuracy rate and error rate are the three most used evaluation criteria. Recall rate indicates the proportion of the target detected in all images; accuracy rate shows the proportion of the target contained in the images detected, and error rate refers to the probability of the target detection error. As iterations increase, the lower the error rate is, the more accurate the recognition result will be. Figure 5a shows the relation curve between the recall rate and the accuracy rate of the detector after the field detector was tested with the test set. When the recall rate was greater than 0.7, the accuracy rate would drop significantly, with the accuracy averaging 0.6. Figure 5b shows the error rate curve of the field object detector. Accompanying the increase of iterations, the error rate of the detector decreased gradually, at an average of 0.5.

**Figure 6:**
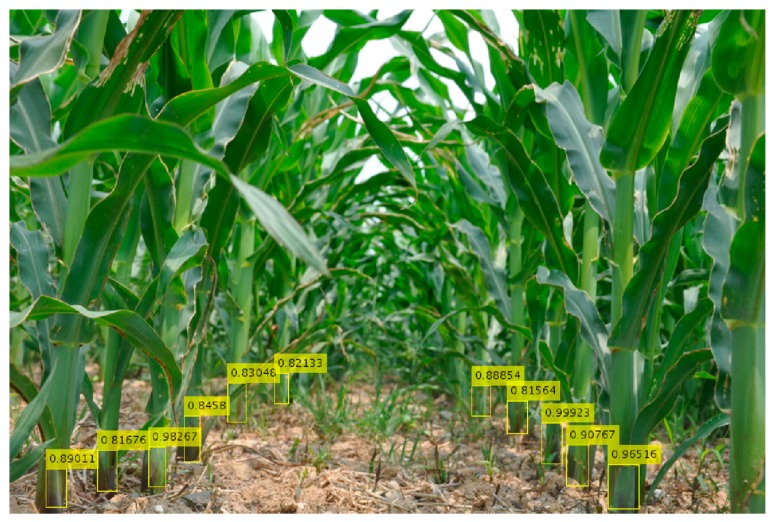
Detection of rhizomes between rows of maize.

Next, we tested the rhizomes of several maize plants in an inter-row environment. Viewing Figure 6, it can be seen that the target rhizome with obvious characteristics can be accurately identified and, even with leaf occlusion, the detection accuracy can be ideally achieved. However, the recognition probability of the model for the distant rhizome was not very high, and some rhizomes were not detected, but the overall recognition effect was quite good.

## 3. Design of Robot Control System

### 3.1. Robot Mathematical Model

The robot involved in this paper mainly performed plant protection operations in the field. Regarding the robot, two front wheels were designed for turning functions and two rear wheels for driving purposes. Concerning terms of direction, the two rear wheels always remained consistent with the robot to establish a 2-DOF robot model. The robot was simplified as follows in modeling:

(1) The plant protection process conducted by the robot was a low-speed movement one, with the speed controlled at 10 km/h.
(2) The robot was capable of only rolling without sliding during the steering.
(3) As the model only took into consideration the motion of a rigid body in a low-speed movement, the robot was not affected by any lateral force in the traveling process, the positioning angle of the front wheel being zero.

**Figure 7:**
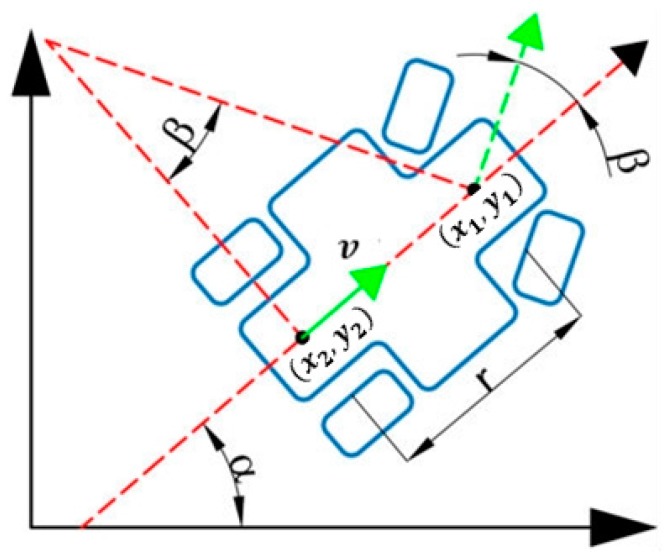
Kinematics Model of Robot α refers to the horizontal angle between the vehicle center line and the coordinate system X axis, β to the turning angle of the robot, r to the distance from the center of the front axle to that of the rear axle of the robot, v to the forward speed of the robot and $\left(x_{1},y_{1}\right),\left(x_{2},y_{2}\right)$ to the center coordinates of the front and rear axles of the robot.

The simplified kinematics model of the robot is shown in Figure 7.

It can be observed from the Figure 7 that the robot met the nonholonomic constraint conditions in the motion process:

(1) $$\dot{y_{2}}cos\alpha -\dot{x_{2}}sin\alpha =0$$

Among them,

(2) $$\{\begin{array}{c} x_{2}=x_{1}-rcos\alpha \\ y_{2}=y_{1}-rsin\alpha \end{array}$$

Derivatives from the formulas are derived as follows:

(3) $$\{\begin{array}{c} \dot{x_{2}}=\dot{x_{1}}+\dot{\alpha }rsin\alpha \\ \dot{y_{2}}=\dot{y_{1}}-\dot{\alpha }rcos\alpha \end{array}$$

Taken from it, we obtained the kinematics equation of the robot based on the center point of the front axle $\left(x_{1},y_{1}\right)$:

(4) $$\{\begin{array}{ll} \dot{x_{1}}= & v\cdot \cos\left(\alpha +\beta \right)\\ \dot{y_{1}}= & v\cdot \sin\left(\alpha +\beta \right)\\ & \dot{\alpha }=\frac{vsin\beta }{r} \end{array}$$

The continuous kinematics equation of the robot could be obtained by introducing (3) into (4):

(5) $$\{\begin{array}{ll} \dot{x_{1}}= & v\cdot \cos\alpha \cdot cos\beta \\ \dot{y_{1}}= & v\cdot \sin\alpha \cdot cos\beta \\ & \dot{\alpha }=\frac{vsin\beta }{r} \end{array}$$

**Figure 8:**
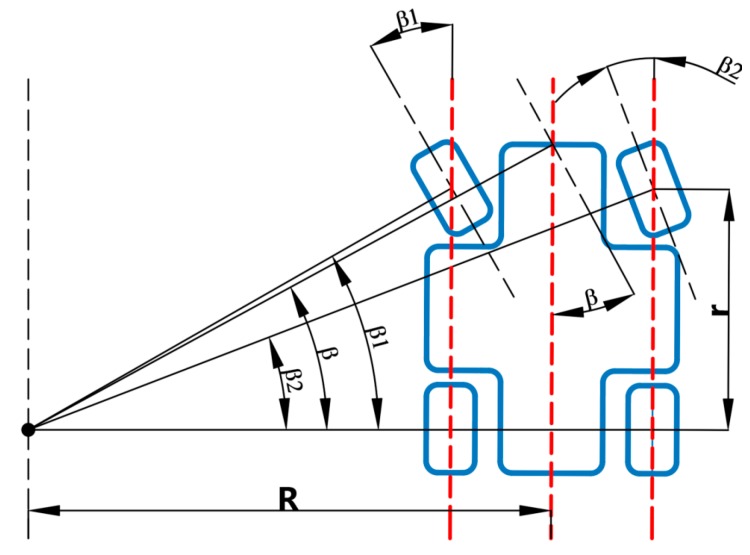
Robot steering diagram. β stands for the turning angle of the robot, β1 for the steering angle of the inner front wheel, and β2 for the steering angle of the outer front wheel.

According to Ackerman’s principle, the steering mechanism of the four-wheeled robot during steering can make the steering angle of the inner wheel 2–4 degrees larger than that of the outer one, so that the center of the trajectory of the four wheels may intersect at a point, which is located on the extension line of the rear axle. Based on Ackerman steering geometry, the kinematics model of robot steering was established, as shown in Figure 8. 

Using Ackerman’s principle, the robot satisfied in the course of steering:

(6) $$\beta =\beta 2+\frac{\beta 1-\beta 2}{2}=\frac{\beta 2+\beta 1}{2}$$

The steering process of the robot involved in this paper was driven by the stepper motor to the steering mechanism rack so the front wheel angle changed to complete steering. Based on the pre-laboratory calibration, the relationship between the motion time of the stepper motor and the steering angle of the front wheel of the robot is roughly shown as follows:

(7) β1=kt

(8) β2=kt−1.5

Thus, it is possible to control the steering of the robot through the regulation of the turning time of the motor.

### 3.2. Control System Design

The state of the robot was represented by the position (x , y) of the center point of the robot axis in the coordinate system and the steering angle β. The ideal trajectory was ($x_{d}\;,$ $y_{d}$) and the ideal steering angle was $\beta_{d},$ which referred to the angle between the ideal direction of the robot and the *X*-axis. To enable fast tracking, the attitude control rate $\omega_{\beta }$ was adopted in this paper, together with the angle β and tracking $\beta_{d}$ implemented, the details of which are shown as follows: 

Take $\beta_{e}=\beta -\beta_{d}$, and the sliding mode function as $s_{3}$ = $\beta_{e}$, then

(9) $$\dot{s_{3}}=\dot{\beta_{e}}=\omega -\dot{\beta_{d}}$$

The design attitude control rate:

(10) $$\omega_{\beta }=\dot{\beta_{d}}-k_{3}s_{3}-\eta_{3}sgns_{3}$$

Among them $k_{3}>0$, $\eta_{3}>0$. 

Then, $\dot{s_{3}}=-k_{3}s_{3}-\eta_{3}sgns_{3}$, take $V_{\beta }=\frac{1}{2}s_{3}{}^{2}\;$, $\dot{V_{\beta }}=s_{3}\dot{s_{3}}=-k_{3}s_{3}{}^{2}-\eta_{3}\left|s_{3}\right|\leq -k_{3}s_{3}{}^{2}\;$, that is to say $\dot{V_{\beta }}\leq -2k_{3}V_{\beta }\;$, thus the angle β exponent converging to $\beta_{d}$. 

According to the literature [3], if the range of $\beta_{d}$ is (−π/2,π/2), the ideal trajectory $\beta_{d}$ can be obtained as:

(11) $$\beta_{d}=\arctan\frac{u_{2}}{u_{1}}$$

Considering the formula: $u_{1}=v\cos\beta ,u_{2}=v\sin\beta$

The output signals of the controller included linear velocity V and angular velocity ω, while the actual input of the robot involved the driving time of the stepping motor t, so the signal converter was designed as follows: make V=v, and $\omega =\omega_{\beta }$.

(12) $$\{\begin{array}{l} \omega_{\beta }\cdot R=v\\ \omega_{\beta }\cdot t=\beta \end{array}$$

The signal converter obtained by formula 12:

(13) $$\{\begin{array}{ll} & t=\frac{1.5}{k-\omega_{\beta }}\\ v= & (k-\frac{1.5}{t})\cdot R \end{array}$$

**Figure 9:**
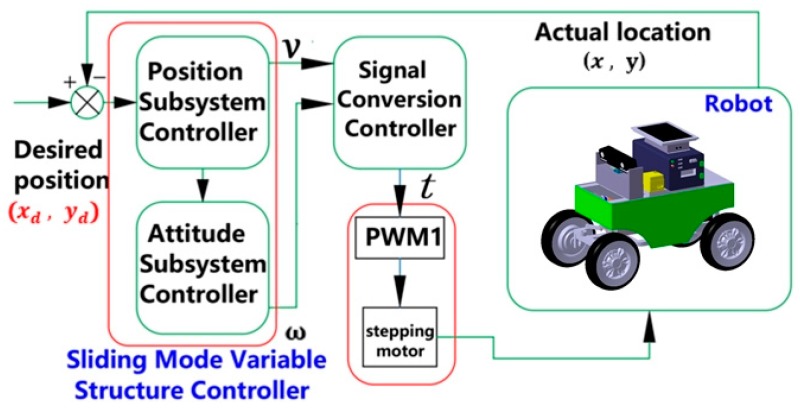
Sliding mode variable structure control strategy.

Thus, the driving of the robot could be controlled by active control t. The control strategy diagram based on the sliding mode variable structure is shown in Figure 9. 

## 4. Path Generation

### 4.1. Path Fitting

To realize autonomous moving and turning in the field, the robot is required not only to recognize the specific positioning point of a corn rhizome, but also to fit the positioning point into the parameters of crop lines. We identified the target rhizomes according to the object detector established above, extracted the fixed point in the regression boundary box as the reference point to form the crop lines, and then used the cubic spline interpolation method to fit the crop lines on both sides of the rhizome. Cubic spline interpolation indicated that there are n points in an interval (a,b), the abscissa being $x_{0}=a<x_{1}\ldots \ldots x_{n\;-\;1}<b=x_{n}$, and the corresponding ordinates being $y_{0},y_{1},\ldots ,y_{n\;-\;1},y_{n}\;$. The two adjacent points $x_{i}\;,$ $x_{i\;+\;1}$ of these n points formed a subinterval $\left[x_{i},x_{i\;+\;1}\right]$, and spline s(x) constituted a subsection defined formula. The cubic spline equation should meet the following requirements: (1) the spline function in each sub interval is an increasing cubic polynomial; (2) the function should have continuity and satisfy the $S\left(x_{i}\right)=y$; and (3) the first and the second derivatives of the spline function are continuous, that is, the spline curve is smooth. The calculation method is provided as follows:

It is assumed that there are n+1 data nodes $\left(x_{0},y_{0}\right),\ldots ,\left(x_{n},y_{0}\right)$:

- Time step:
  (14) $$h_{i}=x_{i\;+\;1}-x_{i}\left(i\;=\;0,1,\ldots ,\;n-1\right)$$
- Substitute the data node and the specified first endpoint condition into the matrix equation:
  (15) $$\left[\begin{array}{cccccc} -h_{1} & h_{0}+h_{1} & -h_{0} & \cdots & \cdots & 0\\ h_{0} & 2\left(h_{0}+h_{1}\right) & h_{1} & 0 & \cdots & \vdots \\ 0 & h_{1} & 2\left(h_{1}+h_{2}\right) & h_{2} & 0 & \vdots \\ \vdots & 0 & \ddots & \ddots & \ddots & 0\\ 0 & \cdots & 0 & h_{n\;-\;2} & 2\left(h_{n\;-\;2}+h_{n\;-\;1}\right) & h_{n\;-\;1}\\ 0 & \cdots & \cdots & -h_{n\;-\;1} & h_{n\;-\;2}+h_{n\;-\;1} & -h_{n\;-\;2} \end{array}\right]$$
- Solve the matrix equation and obtain the quadratic differential value $m_{i}\;$.
- Calculate the coefficient of a spline:
  (16) $$a_{i}=y_{i}$$
  (17) $$b_{i}=\frac{y_{i\;+\;1}-y_{i}}{h_{i}}-\frac{h_{i}}{2}m_{i}-\frac{h_{i}}{6}\left(m_{i\;+\;1}-m_{i}\right)$$
  (18) $$c_{i}=\frac{m_{i}}{2}$$
  (19) $$d_{i}=\frac{m_{i\;+\;1}-m_{i}}{6h_{i}}$$
  where, i=0,1,…,n−1.
- Regarding each subinterval $x_{i}\leq x\leq x_{i\;+\;1}\;$, create the equation
  (20) $$S_{i}\left(x\right)=a_{i}+b_{i}\left(x-x_{i}\right)+c_{i}{\left(x-x_{i}\right)}^{2}+d_{i}{\left(x-x_{i}\right)}^{3}$$

Among them, $a_{i},b_{i},c_{i},\;d_{i}$ represent 4∗n unknown coefficients.

**Figure 10:**
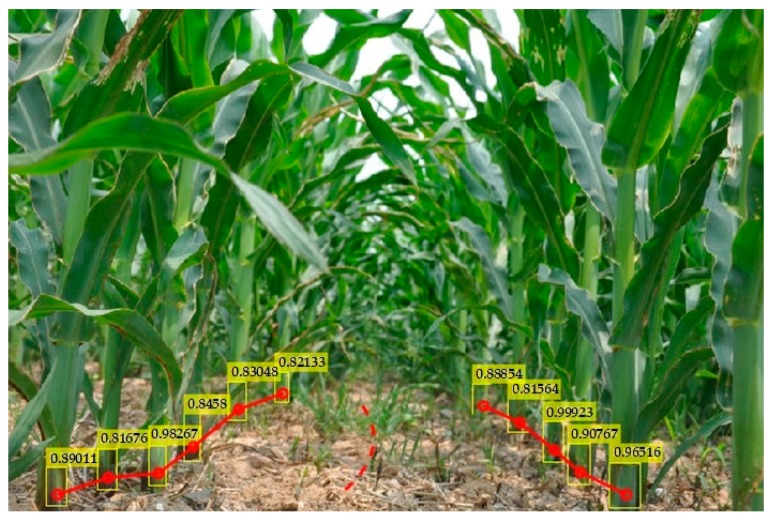
Schematic diagram of line of proposed cooperation.

The crop line between corn lines obtained by this method is shown as a solid line in Figure 10, and the final driving path is shown as a dotted line by fitting with a least square method. Regarding the actual situation, considering the uncertainty of corn germination, there may be a lack of seedlings on one side during the movement of the robot. It can be found, seen in Figure 10, that the robot could recognize five to seven seedlings in front while moving. When the number of missing seedlings is small, the path fitting would not be affected. When there are a greater number of missing seedlings on one side, the path could be planned according to the rhizomes on the other side. The experiment which was conducted in the Huanghuaihai Region, had a row spacing of 60 cm, thus, the position short of seedlings should be supplemented at the corresponding spot 60 cm away from the other side of the rhizome and, then, the path should be fitted according to the original method. 

### 4.2. Coordinate Transformation

**Figure 11:**
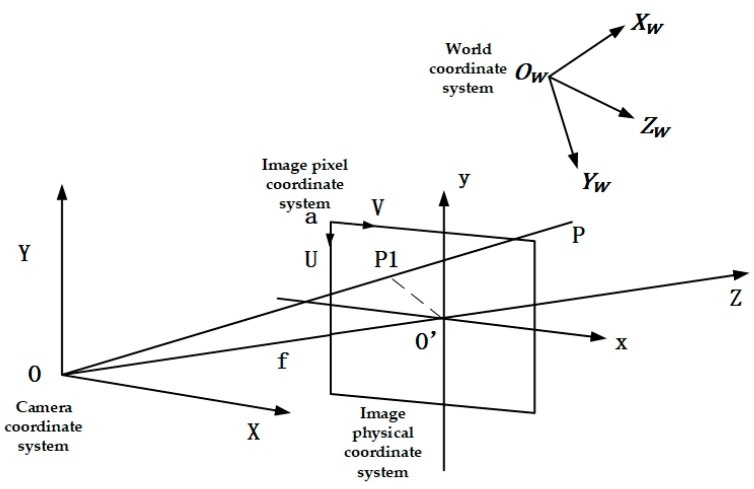
Camera coordinate system diagram.

To know the actual location of coordinate points in the picture, it is necessary to understand the conversional relationship between several coordinate systems. Shown in Figure 11, there are four coordinate systems: the world coordinate system $O_{W}-X_{W}Y_{W}Z_{W}$; the image pixel coordinate system UaV; the image physical coordinate system O’–xy; and the camera coordinate system O–XY.

(1) World coordinate system $O_{W}-X_{W}Y_{W}Z_{W}$

The vertical projection point of the camera’s center O on the ground indicated the origin of the world coordinate system $O_{W}$, and the line between $O_{W}$ and O was taken as the $Z_{W}$ axis of the world coordinate system. Then, the two mutually perpendicular vectors on the ground were taken as the $X_{W}$ and $Y_{W}$ axes of the world coordinate system, and the point P was set as $\left(X_{W},Y_{W},Z_{W}\right)$ in the world coordinate system, which described the actual position of the object.

(2) Image pixel coordinate system UaV

The image pixel coordinate system took the vertex of a frame of image as the origin, and the coordinate system established with image rows and columns is shown in Figure 11.

(3) Image physical coordinate system O’–xy

The geometric center of the image was taken as the origin, and the parallel lines were made parallel to the U-axis and V-axis as the x- and y-axis of the physical coordinate system, respectively. The origin of the physical coordinate was intersected with the z-axis of the camera coordinate system, and its line with the camera coordinate system was coincident with the z-axis. The conversion formula between the image pixel coordinates and the image physical coordinates is given as follows:

(21) $$\{\begin{array}{c} U=\frac{x}{dx}+U_{0}\\ V=\frac{y}{dy}+V_{0} \end{array}$$

where (U,V) refers to the image pixel coordinates, (x,y) to the image physical coordinates, $\left(U_{0},V_{0}\right)$ to the position coordinate value of origin O’of the image physical coordinate system in its image pixel coordinate system, and dx, dy to the physical size of each pixel in the image physical coordinate system.

It is generally more convenient to express and calculate geometric transformations with matrices; thus, the following matrix form was adopted:

(22) $$\left[\begin{array}{c} U\\ V\\ 1 \end{array}\right]=\left[\begin{array}{ccc} \frac{1}{dx} & 0 & U_{0}\\ 0 & \frac{1}{dy} & V_{0}\\ 0 & 0 & 1 \end{array}\right]\left[\begin{array}{c} x\\ y\\ 1 \end{array}\right]$$

(4) Camera coordinate system O−XYZ

Shown in Figure 11, point O is the origin of the camera coordinate system, with its X-axis parallel to the x-axis, its Y-axis to the y-axis, and the Z-axis perpendicular to the image plane. Concerning the camera coordinate system, the 3D homogeneous coordinates of P1 point were (X,Y,Z,1); point P1 was the imaging point of point P in the world coordinate system. According to the relationship of the space transformation model, the transformation formula between them could be obtained as follows:

(23) $$\left[\begin{array}{c} X\\ Y\\ Z\\ 1 \end{array}\right]=\left[\begin{array}{cc} R & T\\ O^{T} & 1 \end{array}\right]\left[\begin{array}{c} X_{W}\\ Y_{W}\\ Z_{W}\\ 1 \end{array}\right]$$

where R is the orthogonal matrix of a 3 ∗ 3 camera rotation transformation, T indicates the attitude matrix of the 3 ∗ 1 order camera translation, and $O^{T}$ is the ${\left(0,0,0\right)}^{T}$.

According to the imaging principle of the camera, the process from the image pixel coordinates to the world coordinates could be calculated, and the following relations thus were obtained:

(24) $$x=f\frac{X}{Z}$$

(25) $$y=f\frac{Y}{Z}$$

where (X,Y,Z) refers to the camera coordinate system coordinates of point P, f to the distance from the origin of the camera coordinate system to the image coordinate system, namely, the focal length of the camera.

The matrix has the expression as follows:

(26) $$Z\left[\begin{array}{c} x\\ y\\ z \end{array}\right]=\left[\begin{array}{cccc} f & 0 & 0 & 0\\ 0 & f & 0 & 0\\ 0 & 0 & 1 & 0 \end{array}\right]\left[\begin{array}{c} X\\ Y\\ Z\\ 1 \end{array}\right]$$

where (x,y,z) are the coordinates of the imaging point P1 of point P in the image plane.

The transformation relationship between the image coordinate system and the world coordinate system could be obtained by solving the formula together as follows:

(27) $$X_{W}=\frac{\left|U-U_{0}\right|*Z_{W}}{f_{x}}$$

(28) $$Y_{W}=\frac{\left|U-U_{0}\right|*Z_{W}}{f_{y}}$$

where $f_{x}\;$, $f_{y}$ refers to the internal parameters of the camera, indicating the focal length in the X- and *Y*-axis directions, and $Z_{W}$ is the depth information measured by the depth camera.

**Figure 12:**
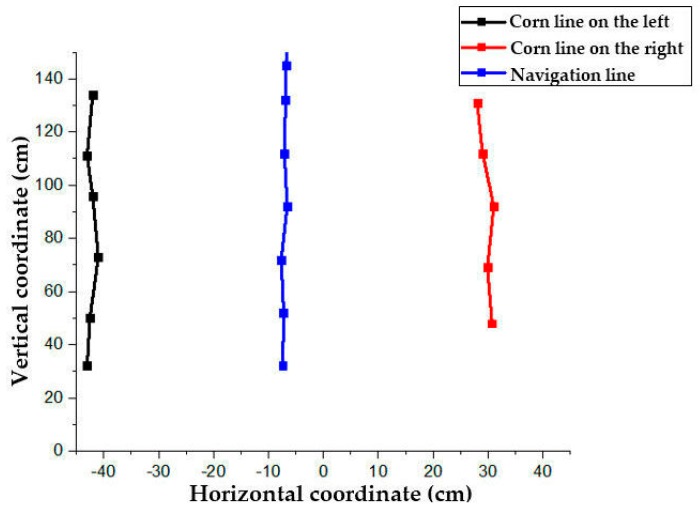
World coordinate system diagram.

Considering the above formula, the conversion between the pixel points of the imaging coordinate system and the actual three-dimensional world coordinate position could be obtained, as shown in Figure 12: 

### 4.3. Robot Motion Control

**Figure 13:**
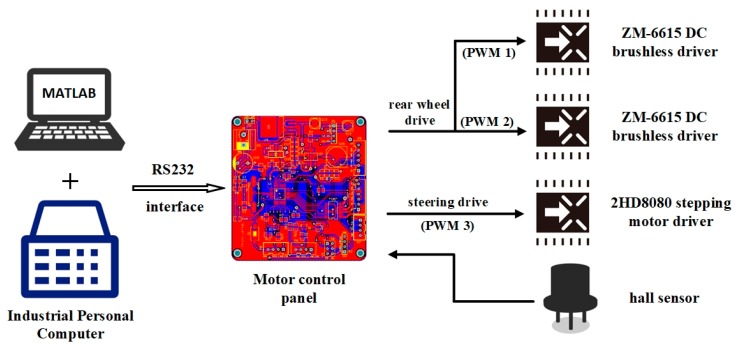
Robot control process.

After the image collected by the camera was transmitted to the computer in real time, the ideal path was fitted by the industrial computer using the trained model, and then the computer sent the robot’s moving control command to the STM32F103C8T6 microcontroller through the recommended standard232 (RS232) interface. The microcontroller sent two identical pulse with modulation (PWM) pulse signals to the signal control end of the ZM-6615 direct current (DC) brushless driver, which was used to drive the two rear wheels of the robot. The robot could move by itself. When the robot went astray, the microcontroller would send a PWM pulse signal to the control end of the 2HD8080 stepping motor driver to adjust the direction. Meanwhile, the hall sensor transmitted the steering angle collected in real time back to the microcontroller for precise steering. The control process is shown in Figure 13. 

## 5. Simulation and Test

### 5.1. Joint Simulation of Path-Following Control

**Figure 14:**
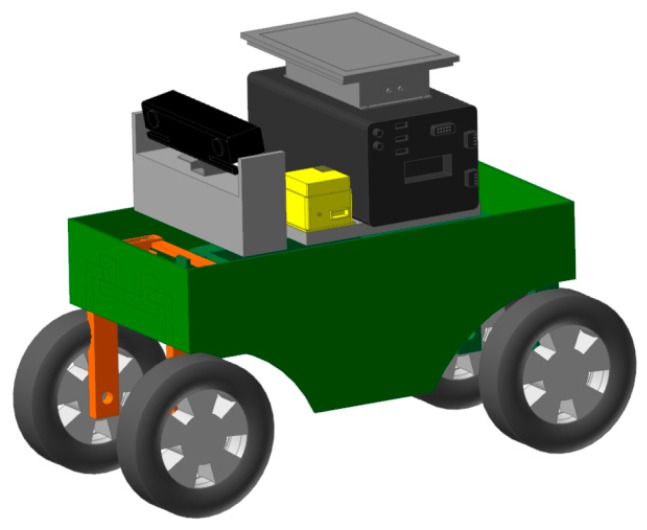
Three dimensional drawing of robot chassis simulation.

After the path tracking control algorithm was designed, the multi-body dynamics simulation software RecurDyn and MATLAB/Simulink were used for the joint control simulation of path tracking. The dynamic model of the robot was established in the software RecurDyn, as shown in Figure 14 (to analyze the robot’s protruding motion system, the non-kinematic parts of the robot were omitted, only the chassis part being reserved, and the parts were regarded as rigid parts except the tires.). 

**Figure 15:**
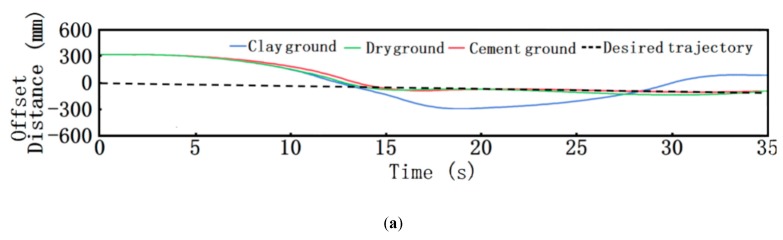

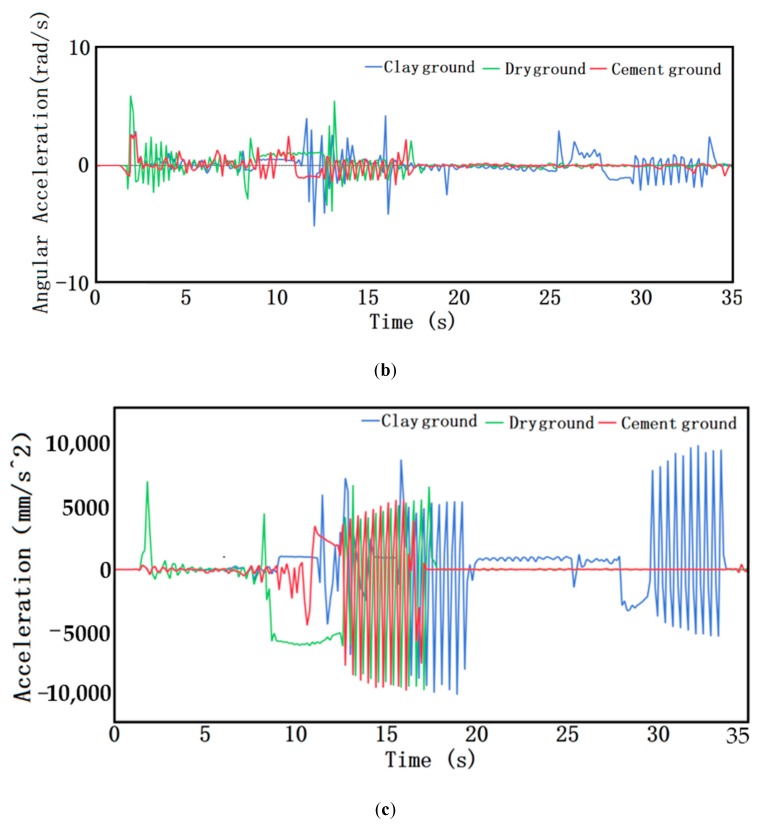
Simulation results: (**a**) Path tracking results of robot on different roads; (**b**) Acceleration diagram of robot’s yaw angle under different road conditions; (**c**) Yaw acceleration diagram of robot under different road conditions.

**Figure 16:**
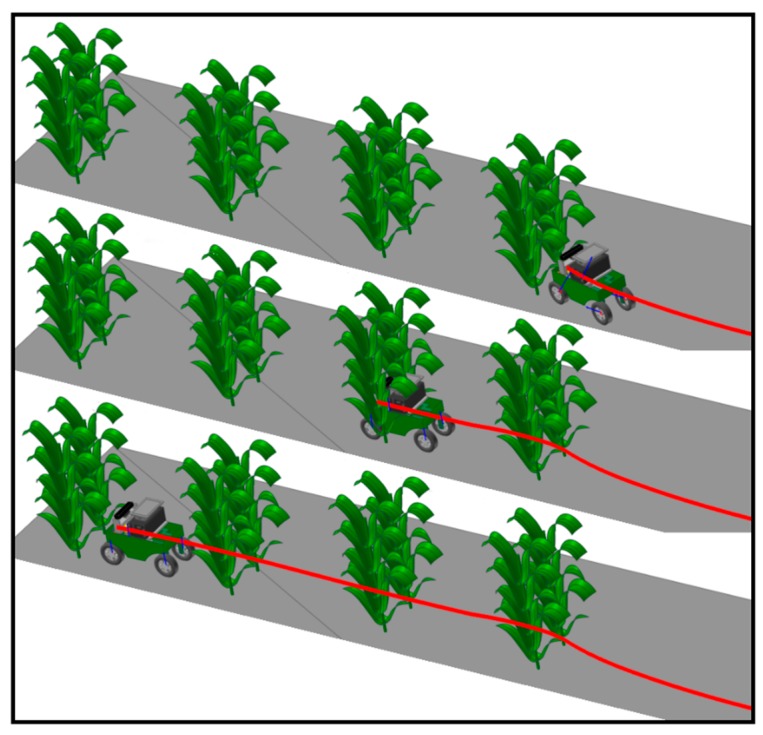
Real time simulation result chart.

During the actual work of the robot, the expected path was obtained by processing the image and fitting the center line of the corn row after the Kinect camera took in the corn row environment. Therefore, the center line was set as the expected path in the joint control simulation of path tracking. Under the premise that the robot traveled at a speed of 0.3 m/s, the simulation conditions of clay, dry soil and cement pavement were selected to analyze the robot’s path tracking performance. The simulation results are shown in Figure 15 and Figure 16. The red curve, blue curve and cyan curve in Figure 15a show the path tracking simulation results of the robot under the conditions of cement road, dry soil and clay respectively. It can be seen from the Figure that, under the condition of the cement road, the path tracking effect of the robot was at its best, followed by the condition of the dry soil, and the robot could not track the desired path well under the condition of the clay due to the tire slipping; viewing Figure 15b, it can be observed that the robot showed very stable yaw acceleration under the conditions of cement pavement and dry soil after tracking the expected path, but the yaw acceleration fluctuated greatly under the conditions of clay; shown in Figure 15c, the robot had a very stable transverse movement under the conditions of cement pavement and dry soil after tracking the expected path, while the transverse displacement speed fluctuated greatly under the condition of clay. It was mainly because the soft clay had a high moisture content, poor adhesion, and high road resistance, which made the stress transfer process to the vehicle at the time very complicated; the driving resistance increased and the adhesion coefficient decreased, which caused the tires to slip and deviate from the originally fitted path. The actual working condition involved the field environment where corn was in the middle and later period of its growth and the road condition was dry soil. According to the simulation results, the robot could track the path stably in such field environment, and the real-time simulation results are shown in Figure 16.

### 5.2. Real-Life Scenario

**Figure 17:**
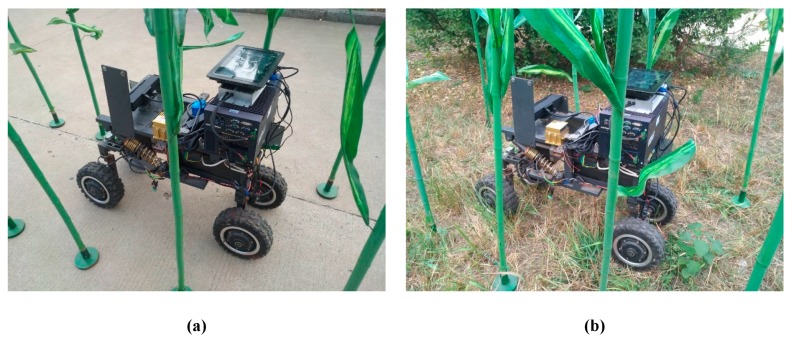
Artificial simulation environment. (**a**) Artificial simulation of road environment; (**b**) Artificial simulation of field environment.

Regarding Anhui Province, China, corn has a growth period generally lasting from May to August. As the experiment was performed in October when the corn had been harvested, the above-mentioned method was verified by setting up an artificial simulated environment for the corn plant experiment. The experiment site was chosen in the Mechanical and Electrical Engineering Park of Anhui Agricultural University. The row and plant spacing were set to 60 cm and 25 cm, respectively, in the simulated experimental environment. The information acquisition robot was required to be able to pass through the corn rows safely at a low speed without hitting the corn plants, as shown in Figure 17. 

**Figure 18:**
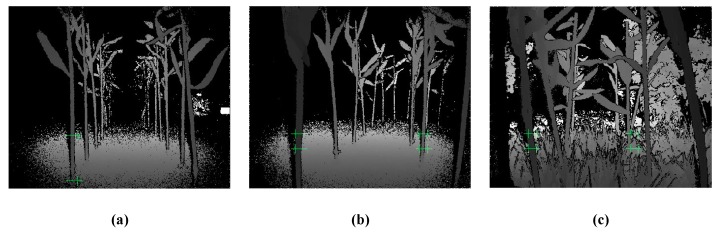
Camera recognition depth map. (**a**) ground straight line; (**b**) ground curve; (**c**) field straight line.

Four types of experimental environments were tested. The depth images of different environments collected by the camera during the experiment are shown in Figure 18. It can be seen that the method proposed in this study could identify the corn rhizome and avoid obstacles when it travelled on the road or in the field. 

**Figure 19:**
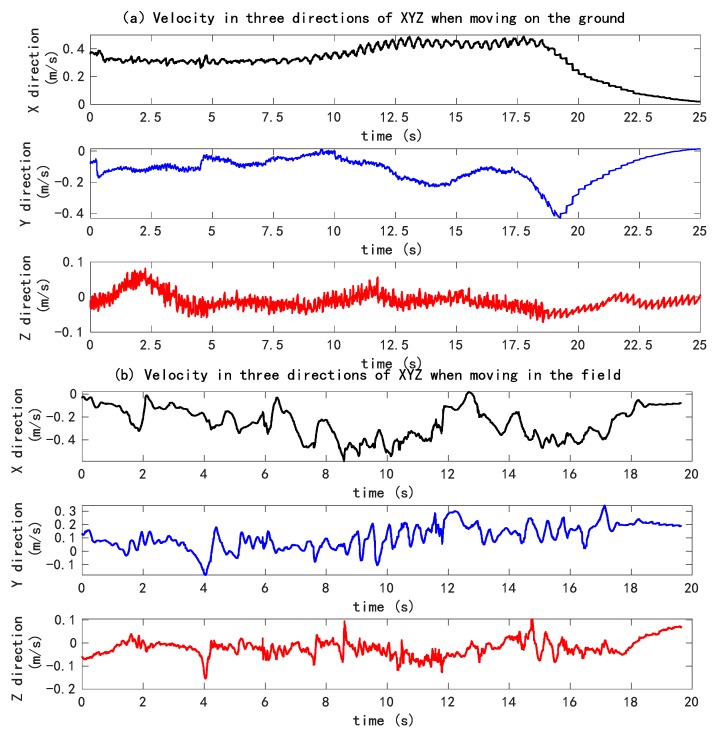
Velocity curve.

During the process of robot motion, the velocity and acceleration values in three directions of XYZ were collected by the inertial navigation installed on the robot, and the curve was simulated as shown in Figure 19, which shows the speed curve of the machine in three directions of XYZ (X stands for the forward direction of the machine, Y for the lateral direction of the machine, and Z for the vertical direction) under the conditions of the cement road and the grass. According to (a) and (b) in Figure 19, the speed change in Z direction was mainly caused by the machine’s action on the ground during driving. Through the comparative analysis of two kinds of pictures, it can be found that the damping components of the machine need to be further optimized. Compared with the cement ground, the grassland has smaller vibration, which is related to the buffering effect of the grassland itself. Shown in Figure 19a, it can be seen that during the process of moving across the ground, the speed in the *Z*-axis direction fluctuated around 0 within a small range, which was related to the fact that the experimental conditions mainly involved straight-line driving at the time, and the speed change was mainly caused by the spacing of the simulated plants on both sides and the lateral force of the road on the vehicle; after 18 s, the speed in X direction decreased too quickly, and the machine entered into the turning state; the corresponding speed in Y direction changed as well. Such a state was caused by the control system for the steering motor after the captured images were processed. Shown in Figure 19b, the significant changes of speed in the X and Y directions were related to the greater interference of weeds in image processing, resulting from the frequent regulation and control of the motor by the control system. Concurrently, the experiment was dominated by complex turning conditions (actual field driving, less weeds, and mainly straight-line driving), which led to the great change in the speed curve.

As the robot moved in the simulated field environment during the experiment, due to the unevenness of the road surface, there were deviations in the direction of the movement, and the deviation from the original fitted path required constant adjustment. However, as there was not much deviation on the cement road, the robot could basically move along the fitted path. Through the comparison of the two results, the complexity of the field environment and the difficulty of the robot moving between lines was made quite clear. 

**Figure 20:**
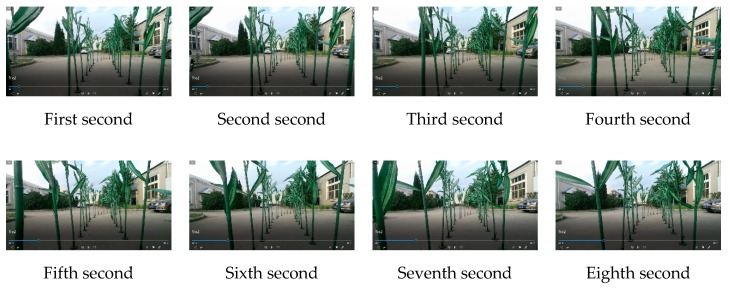

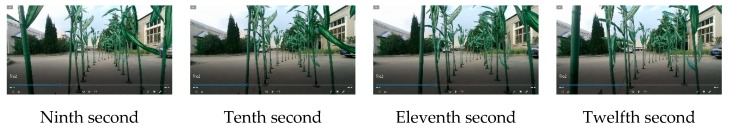
The real-time process during machine movement.

After testing, the latency of this system is currently 0.31 s, the delay mainly determined by the Faster region-CNN (R-CNN) processing speed, control system calculation, and other parts, to reduce latency. High-performance industrial computers are currently used for real-time processing. Figure 20 shows the real-time process during machine movement.

## 6. Discussion and Future Work

Aiming at the issue of plant protection against corn diseases and insect pests, this study designed a robot to collect crop growth information while moving in the field and discussed the feasibility of using the visual sensor to identify corn rhizomes, so that the robot could avoid obstacles and move by itself. Based on Faster region-CNN (R-CNN), a method of target detection, which is based on the visual geometry group-16 (VGG-16) convolution neural network model for migration learning, was proposed. The processing results show that the target rhizomes with obvious characteristics could be accurately identified; even when there was leaf occlusion; it could achieve an ideal accuracy in detection; and the overall detection effect had good results. The robot was driven by a stepping motor; the wheel motor controlled the steering, connecting with the upper computer through a serial port. A control system for a sliding mode variable structure was adopted to identify corn rhizomes through the trained model in combination with the image detection collected by the visual sensor in real time, and the driving path was then fitted. The experiment was carried out on the ground and in the field. As shown by the experimental results, the method was effective and the robot could detect corn rhizomes quickly and avoid obstacles well, in both real-time performance and in the feasibility in the field environment. 

Considering the real environment in Huanghuaihai region, China, the experiment used an artificial simulation environment instead, and those performed under real conditions were during the real growth period. Additionally, the inter-row information during corn growth in the area was collected in the early stage, including information, such as row spacing of 60 cm and plant spacing of 25 cm. The plant and row spacing in the simulated environment was obtained from the real spacing data collected, while the ground environment in the simulation experiment was more complex than the real one since the lines were mostly straight, and the weeds were fewer than those in the simulated environment, so the simulation environment was considered to be representative.

Presently, the research only involved the root image of the middle and later stages of corn growth. Considering future study, the diversity of samples may be increased, other plants such as tobacco be trained, and the method applied to more environments.
